# Characterizing potential subtypes and influencing factors of burnout in emergency department nurses by latent profile analysis

**DOI:** 10.3389/fpubh.2025.1654398

**Published:** 2025-09-25

**Authors:** Lin Lan, Xiaoli Chen, Hao Zhang, LuYing Zhong, Lei Ye

**Affiliations:** ^1^Emergency Department of West China Hospital, Sichuan University, Chengdu, China; ^2^West China School of Nursing, Sichuan University, Chengdu, China; ^3^Institute of Disaster Medicine, Sichuan University, Chengdu, China; ^4^Nursing Department of West China Hospital, Sichuan University, Chengdu, China

**Keywords:** emergency department nurses, burnout, latent profile analysis, influencing factor, cross-sectional study

## Abstract

**Objective:**

This study aims to explore the heterogeneity of burnout among emergency department nurses, identify the factors influencing burnout in different subtypes of emergency nurses, and provide targeted strategies and measures to reduce burnout in emergency department nurses.

**Methods:**

A cross-sectional survey was conducted from December 26, 2023, to January 18, 2024, involving 1,555 emergency nurses from 30 tertiary hospitals in China. The survey was distributed via an online questionnaire platform, which included general demographic information and the Maslach Burnout Inventory (MBI). The collected data were analyzed using latent profile analysis, Kruskal–Wallis *H* test, and multiple logistic regression.

**Results:**

A total of 1,555 questionnaires were sent out in this survey, and finally 1,540 were included for data analysis. The average burnout score among emergency nurses was (4.77 ± 6.16). Occupational burnout could be categorized into three subtypes: low burnout-low professional efficacy (C1), low burnout-high professional efficacy (C2), and high burnout-low professional efficacy (C3). The proportions of these subtypes were 41%, 32.3%, and 26.7%, respectively. Significant differences in the latent categories of burnout were observed for age (χ^2^ = 31.749, *P* < 0.001), education level (χ^2^ = 6.778, *P* = 0.034), professional title (χ^2^ = 21.928, *P* < 0.001), years of work (χ^2^ = 29.269, *P* < 0.001), weekly working hours (χ^2^ = 52.493, *P* < 0.001), number of night shifts (χ^2^ = 34.685, *P* < 0.001), and monthly income (χ^2^ = 18.994, *P* < 0.001).

**Conclusion:**

Occupational burnout is prevalent among emergency nurses, with significant heterogeneity in burnout types. Burnout is associated with age, education level, professional title, number of night shifts, weekly working hours, and monthly income. The heterogeneity of burnout subtypes and their influencing factors provides a basis for future personalized interventions.

## 1 Introduction

With the continuous development of the global healthcare industry and the increasing demand for emergency medical services, emergency nursing is facing unprecedented challenges. Emergency nurses are required to manage high work pressure, heavy tasks, and complex clinical situations (1, 2), while also handling emotional burdens, long shift hours, and sudden patient needs (3). These factors together contribute to the growing prevalence of burnout among emergency nurses (4). Occupational burnout, a common syndrome among professionals, is recognized by the World Health Organization (WHO) as a work-related chronic stress issue in its International Classification of Diseases (5). Burnout affects nurses' physical and mental health (6) and their quality of work life (7), leading to an increased intention to leave the profession (8). Several international studies have shown that nurse burnout is associated with higher patient mortality, infection rates, length of hospital stays, and failure in resuscitation efforts (9, 10). A cross-national study involving six countries found that the higher the nurse burnout, the lower the nursing quality score (11). Therefore, understanding the causes and mechanisms of occupational burnout in emergency nurses has become an important research topic in emergency nursing. Burnout primarily manifests in three core dimensions: emotional exhaustion, depersonalization, and reduced personal accomplishment (12). Emotional exhaustion represents the individual stress dimension of burnout, indicating a state of physical and emotional fatigue resulting from prolonged overuse of emotional resources; it is the most basic and central feature of burnout. Depersonalization represents the interpersonal dimension of burnout, referring to the negative, indifferent, and detached responses individuals show toward work, particularly in relation to their service recipients. Reduced personal accomplishment represents the self-evaluation dimension, indicating a sense of worthlessness, helplessness, and lack of accomplishment in one's work. Although many studies have explored the factors and impacts of burnout (13), there remains a lack of research specifically targeting emergency nurses, especially systematic analyses of the latent factors and characteristics of burnout at different levels. Latent Profile Analysis (LPA) offers significant advantages in identifying latent subgroups within data, handling multidimensional data, avoiding arbitrary classifications, and enhancing classification accuracy. This study aims to conduct a multidimensional latent profile analysis (14) of occupational burnout among emergency nurses, using a quantitative survey to comprehensively explore the latent dimensions and influencing factors of burnout in this group.

## 2 Methods

### 2.1 Study design and participants

This study is part of a cross-sectional survey involving 1,540 emergency nurses in China, focusing on their health status, work-related characteristics, and family dynamics. The survey assessed multiple factors, including somatic symptoms, sleep quality, workplace violence, occupational burnout, work environment, work-family role conflict, and occupational stress among emergency nurses. To date, three articles have been published using this dataset, examining: (a) Gender differences in burnout and work-family conflict among emergency nurses (15); (b) The association between effort-reward imbalance and health outcomes in emergency nurses (16); (c) The relationship between workplace violence and occupational health in emergency nurses (17). This study primarily investigates the latent profile features of occupational burnout in emergency nurses. This research was conducted in accordance with the STROBE guidelines.

### 2.2 Measures

A basic demographic survey designed by the research team was used to collect general sociological information from the 1,555 emergency nurses, including demographic details (gender, age, education level, marital status, childbearing status), lifestyle habits (smoking, alcohol consumption), and work-related characteristics (years of work, weekly working hours, frequency of night shifts, monthly income, etc.).

#### 2.2.1 Occupational burnout survey

The Maslach Burnout Inventory-General Survey (MBI-GS), developed by Maslach et al. (18) and revised by Wei et al. (19), was used to assess the occupational burnout status of the participants. This scale is widely applicable to various occupational groups and has been cross-culturally adapted and validated by domestic scholars, demonstrating good reliability and validity. The scale consists of 16 items, each rated on a 7-point Likert scale (0–6). The questionnaire includes three dimensions: emotional exhaustion, depersonalization, and reduced personal accomplishment. The first two dimensions are scored positively, where higher scores indicate greater burnout; the third dimension is scored reversely, with lower scores indicating greater burnout. The comprehensive burnout score is calculated as follows: Comprehensive burnout score = [0.4 × emotional exhaustion score + 0.3 × depersonalization score + 0.3 × (6 – personal accomplishment score)]. A comprehensive burnout score <1.5 is classified as no burnout, a score between 1.5 and <3.5 as suspected burnout, and a score ≥3.5 as burnout. In this study, the Cronbach's α coefficients for the three dimensions and the overall questionnaire were 0.914, 0.814, 0.899, and 0.860, respectively.

### 2.3 Data collection

An electronic version of the survey was created using the “Wenjuanxing” platform, which included an informed consent form and a link to the questionnaire. Through the Emergency Nursing Specialty Committee of the Chinese Nursing Association, the head nurses of the emergency departments in the participating hospitals were contacted to discuss the research objectives, significance, and procedures. After obtaining consent, the head nurses distributed the survey QR code link to eligible emergency department nurses via WeChat and explained the purpose of the study. Participation was voluntary and anonymous. Each IP address was limited to one response to avoid duplicate submissions. After the questionnaires were collected, they were promptly reviewed, and invalid questionnaires (e.g., those with identical answers, clear patterns, or contradictions) were excluded.

### 2.4 Statistical analysis

Latent profile analysis was performed using Mplus 8.3 software, with the 16-item burnout scores as observed indicators. Profiles were selected from 1 to 5 for analysis. Statistical analysis was conducted using SPSS 25.0. Continuous data were expressed as mean ± standard deviation, while categorical or ordinal data were presented as frequencies, percentages, or proportions. The three latent profiles were treated as dependent variables. One-way ANOVA was used for comparing continuous variables between groups, while categorical variables were analyzed using chi-square tests. Variables showing significant differences were further analyzed using logistic regression, with *P* < 0.05 considered statistically significant.

## 3 Results

### 3.1 Characteristics of participants

A total of 1,540 emergency nurses from 30 hospitals participated in the survey, with an average age of 32.23 ± 6.80 years, and 78.6% were female. The emergency nurses exhibited significant levels of burnout, with an average burnout score of (4.77 ± 6.16). Descriptive analysis of the demographic information, lifestyle habits, and work characteristics of the 1,540 emergency nurses is shown in Table 1.

**Table 1:** Demographic characteristics of emergency nurses.

**Variable**	***n* (%)**
**Age (years)**	
20–29	582 (37.8%)
30–39	734 (47.7%)
40–49	183 (11.9%)
≥50	41 (2.7%)
**Gender**	
Female	1,211 (78.6%)
Male	329 (21.4%)
**Marital status**	
Unmarried	560 (36.4%)
Married	980 (63.6%)
**Fertility status**	
Non-parous	710 (46.1%)
Fertilized	830 (53.9%)
**Education**	
Junior college	186 (12.1%)
Bachelor's degree and above	1,354 (87.9%)
**Job title**	
Primary	907 (58.9%)
Intermediate	574 (37.3%)
Senior	59 (3.8%)
**Years of service (years)**	
≤ 5	491 (31.9%)
6–10	493 (32.0%)
11–15	300 (19.5%)
16–20	121 (7.8%)
≥21	135 (8.8%)
**Total weekly working hours (h)**	
≤ 40	577 (37.5%)
41–48	780 (50.6%)
49–58	123 (8.0%)
≥59	60 (3.9%)
**Night shifts (times/month)**	
0	181 (11.8%)
1–4	239 (15.5%)
5–8	659 (42.8%)
>8	461 (29.9%)
**Monthly income (CNY) (yuan)**	
<4,000	57 (3.7%)
4,000–5,999	149 (9.7%)
6,000–7,999	250 (16.2%)
8,000–9999	368 (23.9%)
≥10,000	716 (46.5%)
**Smoking**	
No	1,453 (94.4%)
Yes	87 (5.6%)
**Drinking**	
No	1,355 (88.0%)
Yes	185 (12.0%)

### 3.2 Descriptive analysis of occupational burnout survey results

The occupational burnout score for emergency nurses was 4.77 ± 6.16. According to the comprehensive burnout score from the burnout scale, a score ≥3.5 is classified as occupational burnout, indicating that emergency nurses generally experience occupational burnout. Emergency nurses scored higher on the first two dimensions and lower on the personal accomplishment dimension, suggesting a higher level of burnout. The average scores for the emotional exhaustion dimension were 11.3 ± 7.76, depersonalization were 8.61 ± 7.58, and personal accomplishment were 13.79 ± 10.04, as shown in Table 2.

**Table 2:** Descriptive analysis of occupational burnout.

**Variable**	**Dimension**	**Minimum**	**Maximum**	**Mean**	**Standard deviation**
Burnout	Emotional exhaustion	0	30	11.30	7.76
	Cynicism	0	30	8.61	7.58
	Professional efficacy	0	36	13.79	10.04
	Total	−9	22.8	4.77	6.16

### 3.3 Latent profile analysis of occupational burnout in emergency nurses

Latent profile analysis was performed using the 16 items of the burnout scale. The number of categories was gradually increased from the initial model, fitting latent profile models for 1–5 categories. The fit indices for each category model are shown in Table 3. The model with one category (zero model) had the worst fit, but as the number of categories increased, the AIC, BIC, and aBIC values consistently decreased. The pLMR and pBLRT indices indicated that the 2-category, 3-category, 4-category, and 5-category models fit well. The reduction in aBIC values became smaller after the 3-category model, suggesting that model improvement slowed down after the third category. Considering all fit indices and clinical relevance, the 3-category model was considered the optimal model.

**Table 3:** Fit indices of the latent profile model for occupational burnout.

**Class**	**AIC**	**BIC**	**aBIC**	**pLMR**	**pBLRT**	**Entropy**	**1**	**2**	**3**	**4**	**5**
1	96335.021	96505.886	96404.230	/	/	/	/	/	/	/	/
2	85483.631	85745.268	85589.607	<0.001	<0.001	0.971	0.723	0.277	/	/	/
3	79358.722	79711.131	79501.465	<0.001	<0.001	0.966	0.410	0.323	0.267	/	/
4	74311.262	74754.444	74490.773	<0.001	<0.001	0.969	0.358	0.361	0.135	0.146	/
5	72100.310	72634.264	72316.588	<0.001	<0.001	0.965	0.334	0.192	0.123	0.261	0.090

AIC, Akaike information criterion; BIC, Bayesian information criterion; aBIC, a djusted BIC; pLMR, Lo-Mendell-Rubin Adjusted Likelihood Ratio Test; pBLRT, Bootstrap Likelihood Ratio Test.

### 3.4 Determining the latent categories of occupational burnout in emergency nurses based on the three-category model

**Figure 1:**
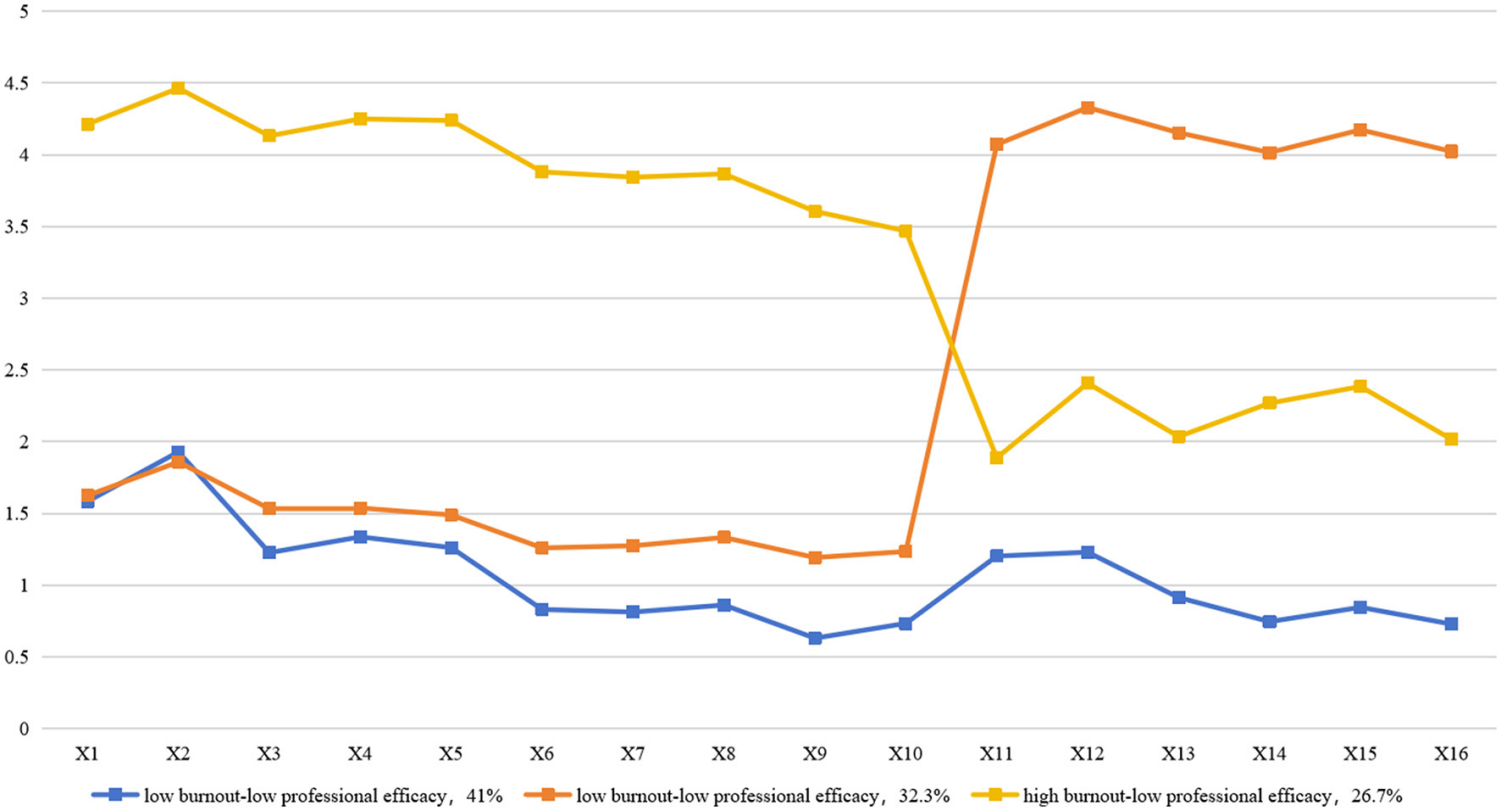
Potential profiles of occupational burnout.

A latent profile graph of emergency nurses' occupational burnout was created using standardized *Z*-scores for emotional exhaustion, depersonalization, and reduced personal accomplishment across the three categories, as shown in Figure 1.

Category C1, which accounted for the highest proportion (41%, 631 nurses), had a burnout profile characterized by low emotional exhaustion, low depersonalization, and low professional efficacy. Category C2, which made up 32.3% (497 nurses), had a burnout profile characterized by low emotional exhaustion, low depersonalization, and high professional efficacy. Category C3, which represented 26.7% (412 nurses), had a burnout profile characterized by high emotional exhaustion, high depersonalization, and low professional efficacy.

These categories were named “low burnout-low professional efficacy,” “low burnout-high professional efficacy,” and “high burnout-low professional efficacy” based on their score characteristics.

### 3.5 Analysis of factors influencing latent profiles

#### 3.5.1 Univariate analysis of demographic variables

The results of the univariate analysis showed that there were significant differences between the latent categories of nurses in terms of age (χ^2^ = 31.749, *P* < 0.001), education level (χ^2^ = 6.778, *P* = 0.034), job title (χ^2^ = 21.928, *P* < 0.001), years of work experience (χ^2^ = 29.269, *P* < 0.001), weekly working hours (χ^2^ = 52.493, *P* < 0.001), frequency of night shifts (χ^2^ = 34.685, *P* < 0.001), and monthly income (χ^2^ = 18.994, *P* < 0.001), as shown in Table 4.

**Table 4:** Single-factor analysis of the profile types of occupational burnout.

**Variable**	**C1**	**C2**	**C3**	***χ^2^***	***P***
**Age (years)**				31.749	**<0.001**
20–29	242 (38.3%)	208 (41.9%)	132 (32.1%)		
30–39	275 (43.5%)	239 (48.1%)	220 (53.5%)		
40–49	86 (13.6%)	44 (8.9%)	53 (12.9%)		
≥50	29 (4.6%)	6 (1.2%)	6 (1.5%)		
**Gender**				2.212	0.331
Female	508 (80.4%)	388 (78.1%)	315 (76.6%)		
Male	124 (19.6%)	109 (21.9%)	96 (23.4%)		
**Marital status**				6.002	0.050
Unmarried	224 (35.4%)	201 (40.4%)	135 (32.8%)		
Married	408 (64.6%)	296 (59.6%)	276 (67.2%)		
**Fertility status**				4.269	0.118
Non-parous	279 (44.1%)	248 (49.9%)	183 (44.5%)		
Fertilized	353 (55.9%)	249 (50.1%)	228 (55.5%)		
**Education**				6.778	**0.034**
Junior college	83 (13.1%)	68 (13.7%)	35 (8.5%)		
Bachelor's degree and above	549 (86.9%)	429 (86.3%)	376 (91.5%)		
**Job title**				21.928	**<0.001**
Primary	383 (60.6%)	314 (63.2%)	210 (51.1%)		
Intermediate	221 (35.0%)	175 (35.2%)	178 (43.3%)		
Senior	28 (4.4%)	8 (1.6%)	23 (5.6%)		
**Years of service (years)**				29.269	**<0.001**
≤ 5	214 (33.9%)	172 (34.6%)	105 (25.5%)		
6–10	180 (28.5%)	173 (34.8%)	140 (34.1%)		
11–15	114 (18.0%)	92 (18.5%)	94 (22.9%)		
16–20	50 (7.9%)	33 (6.6%)	38 (9.2%)		
≥21	74 (11.7%)	27 (5.4%)	34 (8.3%)		
**Total weekly working hours (h)**				52.493	**<0.001**
≤ 40	263 (41.6%)	204 (41.0%)	110 (26.8%)		
41–48	314 (49.7%)	247 (49.7%)	219 (53.3%)		
49–58	38 (6.0%)	35 (7.0%)	50 (12.2%)		
≥59	17 (2.7%)	11 (2.2%)	32 (7.8%)		
**Night shifts (times/month)**				34.685	**<0.001**
0	89 (14.1%)	52 (10.5%)	40 (9.7%)		
1–4	105 (16.6%)	91 (18.3%)	43 (10.5%)		
5–8	266 (42.1%)	228 (45.9%)	165 (40.1%)		
>8	172 (27.2%)	126 (25.4%)	163 (39.7%)		
**Monthly income (CNY) (yuan)**				18.994	**<0.001**
<4,000	16 (2.5%)	20 (4.0%)	21 (5.1%)		
4,000–5,999	51 (8.1%)	54 (10.9%)	44 (10.7%)		
6,000–7,999	86 (13.6%)	88 (17.7%)	76 (18.5%)		
8,000–9,999	153 (24.2%)	124 (24.9%)	91 (22.1%)		
≥10,000	326 (51.6%)	211 (42.5%)	179 (43.6%)		

Bold font indicates a *p*-value <0.05, representing a statistically significant difference.

#### 3.5.2 Multivariate logistic regression analysis

Multivariate logistic regression was performed to analyze the factors influencing the latent burnout profiles. Table 5 shows that, compared with the “low burnout-low professional efficacy” (C1) nurses, those aged 20–29 years (*p* = 0.030) and with a monthly income ≥10,000 RMB compared to those earning <4,000 RMB (*p* = 0.049, 4,000–5,999 RMB *p* = 0.019, 6,000–7,999 RMB *p* = 0.021) were more likely to experience “low burnout-high professional efficacy” (C2). Compared with C3 (“high burnout-low professional efficacy”), those aged 20–29 years (*p* = 0.009), 30–39 years (*p* = 0.012), and 40–49 years (*p* = 0.031) were more likely to belong to the C3 burnout category than those aged ≥50 years. Monthly income (*p* < 0.05) also played a role, with lower income (<4,000 RMB) associated with higher likelihood of C1 burnout. Primary job titles were more likely to be associated with C1 (*p* = 0.034), while fewer night shifts (0 times *p* = 0.008, 1–4 times *p* = 0.001) were more likely to lead to C1 burnout. Longer weekly working hours (>59 h) increased the likelihood of C3 burnout.

**Table 5:** Multivariate regression results of the profile types of occupational burnout.

**Reference group: C1**		***B***	***P***	**Exp(B)**	**95%CI**	
					**Lower limit**	**Upper limit**
C2	Intercept	−2.579	0.000			
	Age (years)reference group: ≥50					
	20–29	1.497	**0.030**	4.470	1.158	17.251
	30–39	1.223	0.056	3.399	0.970	11.907
	40–49	0.809	0.126	2.246	0.797	6.330
	Education reference group: Bachelor's degree and above					
	Junior college	0.125	0.506	1.133	0.784	1.636
	Job title reference group: Senior					
	Primary	0.411	0.375	1.508	0.609	3.737
	Intermediate	0.569	0.199	1.767	0.741	4.216
	Years of service (years) reference group: ≥21					
	≤ 5	−0.220	0.681	0.802	0.281	2.290
	6–10	0.257	0.589	1.294	0.508	3.295
	11–15	0.161	0.728	1.175	0.474	2.914
	16–20	0.230	0.544	1.259	0.599	2.646
	Total weekly working hours (h) reference group: ≥59					
	≤ 40	0.261	0.522	1.299	0.584	2.890
	41–48	0.272	0.502	1.313	0.593	2.904
	49–58	0.445	0.337	1.560	0.630	3.862
	Night shifts (times/month) reference group: 8					
	0	0.125	0.596	1.134	0.713	1.804
	1–4	0.167	0.379	1.182	0.815	1.714
	5–8	0.141	0.351	1.151	0.856	1.547
	Monthly income (CNY) (yuan)reference group: ≥10,000					
	<4,000	0.712	**0.049**	2.039	1.003	4.141
	4,000–5,999	0.524	**0.019**	1.689	1.090	2.618
	6,000–7,999	0.421	**0.021**	1.524	1.066	2.179
	8,000–9,999	0.164	0.292	1.178	0.868	1.599
C3	Intercept	0.034	0.957			
	Age (years) reference group: ≥50					
	20–29	1.836	**0.009**	6.270	1.595	24.641
	30–39	1.600	**0.012**	4.951	1.412	17.362
	40–49	1.146	**0.031**	3.147	1.112	8.906
	Education reference group reference group: Bachelor's degree and above					
	Junior college	−0.331	0.152	0.719	0.457	1.130
	Job title reference group: senior					
	Primary	−0.811	**0.034**	0.444	0.210	0.940
	Intermediate	−0.365	0.301	0.694	0.348	1.387
	Years of service (years) reference group: ≥21					
	≤ 5	−0.941	0.086	0.390	0.133	1.143
	6–10	−0.306	0.521	0.737	0.290	1.873
	11–15	−0.306	0.505	0.736	0.300	1.810
	16–20	−0.114	0.759	0.892	0.431	1.849
	Total weekly working hours (h) reference group: ≥59					
	≤ 40	−1.269	**0.000**	0.281	0.146	0.543
	41–48	−0.805	**0.014**	0.447	0.236	0.848
	49–58	−0.305	0.429	0.737	0.346	1.569
	Night shifts (times/month)reference group:>8					
	0	−0.679	**0.008**	0.507	0.308	0.836
	1–4	−0.735	**0.001**	0.480	0.312	0.737
	5–8	−0.298	0.053	0.742	0.548	1.004
	Monthly income (CNY) (yuan)reference group:≥10,000					
	<4,000	1.067	**0.005**	2.905	1.387	6.086
	4,000–5,999	0.672	**0.005**	1.959	1.221	3.142
	6,000–7,999	0.575	**0.003**	1.776	1.214	2.600
	8,000–9,999	0.121	0.479	1.129	0.807	1.579

C1, low burnout-low professional efficacy group; C2, low burnout-high professional efficacy group; C3, high burnout-low professional efficacy group. Bold font indicates a *p*-value <0.05, representing a statistically significant difference.

## 4 Discussion

### 4.1 Profile characteristics of occupational burnout in emergency nurses

Emergency nurses generally experience occupational burnout, consistent with studies by other researchers (20, 21). The occupational burnout of emergency nurses is categorized into three types. The “low burnout—low professional efficacy” group (C1) accounts for 41%, which is the largest group. This group has low scores in emotional exhaustion and depersonalization, indicating that these nurses experience less psychological fatigue and energy depletion, maintain a high level of responsibility toward patients, and do not show indifference or negative attitudes. However, their professional efficacy score is low, suggesting a lack of confidence in their professional abilities and potentially low self-efficacy. The “low burnout—high professional efficacy” group (C2) accounts for 32.3%, indicating that some emergency nurses, despite facing pressure, still maintain high confidence in their professional abilities. Their emotional exhaustion and depersonalization scores are low, showing relatively light psychological burdens and fatigue, while their professional efficacy score is high, demonstrating full recognition of their professional value and abilities. The “high burnout—low professional efficacy” group (C3) is the smallest at 26.7%, and this group requires special attention. It exhibits the most prominent burnout characteristics, with high scores in emotional exhaustion and depersonalization, indicating that these nurses have long endured high levels of work pressure and their psychological energy is depleted. They may display negative attitudes toward work and patients, and their low professional efficacy score suggests a lack of self-confidence, potentially doubting their professional abilities.

### 4.2 Factors influencing occupational burnout in emergency nurses

#### 4.2.1 Demographic characteristics

Age: There is a strong relationship between the age of emergency nurses and occupational burnout (22). When comparing C1 (“low burnout—low professional efficacy”) with C2 (“low burnout—high professional efficacy”), nurses aged 20–29 are more likely to belong to the C2 burnout group, as younger nurses are more likely to exhibit low burnout but high professional efficacy. This may be because younger nurses, who are new to the workforce, experience some adjustment stress but perform well in basic clinical tasks, leading to high professional efficacy (23). When comparing C1 (“low burnout—low professional efficacy”) with C3 (“high burnout—low professional efficacy”), nurses younger than 39 are more likely to experience C3 burnout, with the likelihood of C3 decreasing with age. Middle-aged nurses tend to be more mature in terms of skills, experience, and career adaptation. However, they may face accumulated work pressure, family responsibilities, and career development bottlenecks, which increase emotional exhaustion while still maintaining some level of professional efficacy. As age increases (24), psychological resilience and professional skills improve, and older nurses tend to handle work pressure and emotional fluctuations better. When disregarding professional efficacy, nurses' occupational burnout demonstrates an inverted U-shaped trajectory across the lifespan: it progressively increases with age, peaks during middle adulthood, and subsequently declines thereafter. This phenomenon occurs because middle-aged nurses face triple challenges—career plateau, family caregiving burdens, and declining physical stamina—leading to peak burnout levels, while senior nurses demonstrate gradual burnout reduction through mature adaptation strategies and work adjustments, ultimately forming the characteristic inverted U-shaped curve. A comparison between Group C1 and Group C3 revealed that younger nurses are more prone to type C3 job burnout. Since the number of nurses aged 20–29 far exceeds those over 40, a larger proportion of emergency nurses fall into the C3 category of burnout, characterized by high burnout and low efficacy. Nursing administrators should take measures to enhance nurses' efficacy and reduce job burnout based on the factors influencing it. Education: Multiple studies have shown that education level is a factor influencing nurse burnout (25, 26). Nurses with higher education may have higher career expectations while facing more professional challenges. Higher education is often associated with greater work burden and pressure, such as taking on more management responsibilities or engaging in academic research. Furthermore, highly educated nurses may have higher expectations for their career development, and failure to meet these expectations may lead to disappointment and burnout.

#### 4.2.2 Work characteristics

Professional Title: Nurses with lower professional titles tend to have less experience (27) and face higher technical demands, leading to greater stress. However, because they still have high expectations for their career, they may try harder to adapt and reduce some of the burnout. Nurses with a lower professional title are more likely to belong to the C1 burnout group, suggesting that the higher the professional title, the less likely a nurse is to experience high burnout (28). Senior nurses typically have more clinical experience and professional accumulation, and are more involved in management work, with less direct involvement in high-intensity clinical tasks. They have higher decision-making power and autonomy, allowing them to better control their work pace and reduce burnout. Nurses with higher professional titles may have relatively higher incomes in the hospital, receive greater recognition from colleagues and superiors, and experience a stronger sense of personal achievement, leading to a significant reduction in job burnout. However, senior nurses, who have been in high-load, high-responsibility positions for extended periods, may experience both psychological and physical fatigue. Years of Work Experience: Work experience is usually correlated with a nurse's age. Nurses with shorter work experience (1–5 years) are still in the career adjustment phase and are more likely to experience high burnout, with clear emotional exhaustion, a lack of experience and skills, and low self-confidence, making them more susceptible to work-related stress. Nurses with medium work experience (6–15 years) often handle a large volume of clinical tasks but are in a stable period of their careers, having accumulated rich experience, which enhances their adaptability and alleviates burnout, though they may face career development bottlenecks. Nurses with long work experience (≥16 years) may experience burnout due to the repetitive nature of their work or health issues, while also lacking motivation for further career development (29). Work Hours: Nurses with shorter weekly working hours ( ≤ 40 h) are less likely to experience C3 burnout and tend to have lower burnout levels. Conversely, longer work hours increase the likelihood of high burnout. Work hours (30) are an important factor influencing burnout. Emergency nurses, who work at a fast pace and have heavy workloads, are more likely to experience burnout due to prolonged periods of high-intensity and high-pressure work, leading to both physical fatigue and psychological stress. Night Shifts: Nurses working more than eight night shifts per month are more likely to experience C3 burnout. Frequent night shifts disrupt the nurse's circadian rhythm, which may lead to sleep disorders, emotional instability, and reduced work efficiency, all of which are important contributors to burnout (31, 32). Emergency nurses must work 24-h shifts to ensure timely patient care, and night shifts are often accompanied by emergencies and urgent tasks, increasing the physical and psychological demands placed on nurses. Income Level: When comparing C1 with C2, higher income levels (≥10,000 yuan) are associated with the C2 burnout group, while lower incomes (e.g., <4,000 yuan) increase the likelihood of belonging to C1. This suggests that higher income is linked to low burnout and high professional efficacy. A higher income (21) gives nurses a sense of fair compensation and work recognition, enhancing their professional identity and motivation. Conversely, lower income may make nurses feel undervalued, contributing to burnout. Nurses with low incomes may also experience financial stress, which further exacerbates burnout risks. Low salary, high work pressure, and limited career advancement opportunities can add to nurses' psychological burdens.

### 4.3 Limitation

While the latent profile analysis identified three distinct burnout subtypes among ED nurses, several limitations should be noted. First, sample constraints, participants were recruited solely from China EDs, limiting generalizability to other countries or healthcare systems. Second, cross-sectional nature, the static classification cannot capture dynamic transitions between subtypes. Longitudinal validation is warranted. Variable selection: Profiles were constructed based on emotional exhaustion, cynicism, professional efficacy, while potentially influential factors (e.g., organizational support, personality traits) were unaccounted for. Third, methodological considerations: LPA results are sensitive to indicator selection and fit criteria (e.g., BIC/ABIC). Replication with alternative methods is recommended. Lastly, Clinical applicability: Translating statistical subtypes into actionable clinical identification remains challenging.

## 5 Conclusion

This study identified three latent profile types of occupational burnout in emergency nurses: “low burnout-low professional efficacy,” “low burnout-high professional efficacy,” and “high burnout-low professional efficacy.” Significant correlations were found between these burnout types and factors such as age, education, job title, night shift frequency, working hours, and monthly income. The findings provide a scientific basis for reducing burnout in emergency nurses, including strategies like optimizing shift schedules (33), improving work environments, offering mindfulness training (34, 35), and providing better technical and medical support.

## Data Availability

The original contributions presented in the study are included in the article/supplementary material, further inquiries can be directed to the corresponding author.

## References

[1] Ilić IM Arandjelović MŽ Jovanović JM Nešić MM. Relationships of work-related psychosocial risks, stress, individual factors and burnout - questionnaire survey among emergency physicians and nurses. Med Pr. (2017) 68:167–78. 10.13075/mp.5893.00516 28345677

[2] Rozo JA Olson DM Thu HS Stutzman SE. situational factors associated with burnout among emergency department nurses. Workplace Health Saf . (2017) 65:262–5. 10.1177/2165079917705669 28557637

[3] Power H Skene I Murray E. The positives, the challenges and the impact; an exploration of early career nurses experiences in the Emergency Department. Int Emerg Nurs. (2022) 64:101196. 10.1016/j.ienj.2022.101196 36108493

[4] Adriaenssens J De Gucht V Maes S. Determinants and prevalence of burnout in emergency nurses: a systematic review of 25 years of research. Int J Nurs Stud. (2015) 52:649–61. 10.1016/j.ijnurstu.2014.11.004 25468279

[5] World Health Organization. International Statistical Classification of Diseases and Related Health Problems (ICD). Geneva: World Health Organization (2019). Available online at: https://icd.who.int/

[6] Hall LH Johnson J Watt I Tsipa A O'Connor DB. Healthcare staff wellbeing, burnout, and patient safety: a systematic review. PLoS ONE. (2016) 11:e0159015. 10.1371/journal.pone.0159015 27391946 PMC4938539

[7] Wang QQ Lv WJ Qian RL Zhang YH. Job burnout and quality of working life among Chinese nurses: a cross-sectional study. J Nurs Manag. (2019) 27:1835–44. 10.1111/jonm.12884 31571326

[8] Ma Y Chen F Xing D Meng Q Zhang Y. Study on the associated factors of turnover intention among emergency nurses in China and the relationship between major factors. Int Emerg Nurs. (2022) 60:101106. 10.1016/j.ienj.2021.101106 34864323

[9] Schlak AE Aiken LH Chittams J Poghosyan L McHugh M. Leveraging the work environment to minimize the negative impact of nurse burnout on patient outcomes. Int J Environ Res Public Health. (2021) 18:610. 10.3390/ijerph18020610 33445764 PMC7828279

[10] Cimiotti JP Aiken LH Sloane DM Wu ES. Nurse staffing, burnout, and health care-associated infection. Am J Infect Control. (2012) 40:486–90. 10.1016/j.ajic.2012.02.029 22854376 PMC3509207

[11] Poghosyan L Clarke SP Finlayson M Aiken LH. Nurse burnout and quality of care: cross-national investigation in six countries. Res Nurs Health. (2010) 33:288–98. 10.1002/nur.20383 20645421 PMC2908908

[12] Maslach C. and Leiter MP, Understanding the burnout experience: recent research and its implications for psychiatry. World Psychiatry. (2016) 15:103–11. 10.1002/wps.20311 27265691 PMC4911781

[13] Nyarko BA Yin Z Chai X Yue L. Nurses' alarm fatigue, influencing factors, and its relationship with burnout in the critical care units: a cross-sectional study. Aust Crit Care. (2024) 37:273–80. 10.1016/j.aucc.2023.06.010 37580238

[14] Qiu HZ Latent class Model: Principles and Techniques. Beijing: Educational Science Publishing House (2008).

[15] Diao D Chen X Zhong L Zhang H Zhang J. Sex differences in burnout and work-family conflict among Chinese emergency nurses: a cross-sectional study. Front Public Health. (2024) 12:1492662. 10.3389/fpubh.2024.1492662 39712298 PMC11659251

[16] Tong L Zhu L Zhang H Zhong L Diao D Chen X. Effort-reward imbalance and health outcomes in emergency nurses: the mediating role of work-family conflict and intrinsic effort. Front Public Health. (2024) 12:1515593. 10.3389/fpubh.2024.1515593 39830181 PMC11740724

[17] Zhang H Zhou J Zhong L Zhu L Chen X. Relationship between workplace violence and occupational health in emergency nurses: the mediating role of dyssomnia. Nurs Crit Care. (2025) 30:e70008. 10.1111/nicc.70008 40075212

[18] Maslach C Schaufeli WB Leiter MP. Job burnout. Annu Rev Psychol. (2001) 52:397–422. 10.1146/annurev.psych.52.1.397 11148311

[19] Zhu W Lou XP Wang ZM. To evaluate the reliability and validity of Maslash Burnout Inventory generic version in nurses. Chin Behav Med Sci. (2007) 16:849–51. 10.3760/cma.j.issn.1674-6554.2007.09.031

[20] Moscu CA Marina V Dragomir L Anghele AD Anghele M. The impact of burnout syndrome on job satisfaction among emergency department nurses of emergency clinical county hospital “Sfântul Apostol Andrei” of Galati, Romania. Medicina. (2022) 58:1516. 10.3390/medicina58111516 36363475 PMC9698052

[21] Munn LT O'Connell N Huffman C McDonald S Gibbs M Miller C. Job-related factors associated with burnout and work engagement in emergency nurses: evidence to inform systems-focused interventions. J Emerg Nurs. (2024) 51249–60. 10.1016/j.jen.2024.10.007 39530969 PMC11885018

[22] Udho S Kabunga A. Burnout and associated factors among hospital-based nurses in Northern Uganda. Biomed Res Int. (2022) 24:8231564. 10.1155/2022/8231564 35372575 PMC8970891

[23] Almezieny T Alashaikh A Alnasser R Dammas S Alsubaie N Alhusaini Y. Prevalence and impact of burnout in Emergency Department nurses; a multicenter study in Riyadh, Saudi Arabia. Med Sci. (2024) 28:1–12. 10.54905/disssi.v28i145.e14ms3304

[24] Yu X Qu H Ni Y Tang X Zhou H. Comparative study of job burnout in Shanghai-based nurses against Maslach norm and Hangzhou norm. Int Nurs Rev. (2025) 72:e12952. 10.1111/inr.12952 38708847

[25] Xie J Li J Wang S Li L Wang K Duan Y. Job burnout and its influencing factors among newly graduated nurses: a cross-sectional study. J Clin Nurs. (2021) 30:508–17. 10.1111/jocn.15567 33205476

[26] Zhang XM Liu Y Xu Y. Investigation and analysis of job burnout of nurses in emergency department. Gen Pract Nurs. (2011) 9:1023–4. 10.3969/j.issn.1674-4748.2011.011.060

[27] You JH Dong HM. Investigation on burnout and work engagement of emergency nurses in 8 secondary hospitals in Ningbo. Hosp Stat China. (2017) 24:144–7. 10.3969/j.issn.1006-5253.2017.02.023

[28] Chen LH. Investigation and analysis of job burnout of nurses in emergency department. Int J Nurs. (2013) 32:561–3. 10.3760/cma.j.issn.1673-4351.2013.03.062

[29] Liu C. Relationship between life style and job burnout of nurses in emergency department in Luohe city. J Xinxiang Med Coll. (2013) 285–7.

[30] Cochran KL Doo K Squires A Shah T Rinne S Mealer M. addressing burnout syndrome from a critical care specialty organization perspective. AACN Adv Crit Care. (2020) 31:158–66. 10.4037/aacnacc2020579 32525998

[31] Bagheri Hosseinabadi M Ebrahimi MH Khanjani N Biganeh J Mohammadi S Abdolahfard M. The effects of amplitude and stability of circadian rhythm and occupational stress on burnout syndrome and job dissatisfaction among irregular shift working nurses. J Clin Nurs. (2019) 28:1868–78. 10.1111/jocn.14778 30653765

[32] Li L Wang X Zhou J Liu M Wang S Zhou Y. Factors associated with chronotype, job burnout, and perceived stress among nurses in Chinese tertiary hospitals: a multicenter cross-sectional study. Chronobiol Int. (2024) 41:1058–67. 10.1080/07420528.2024.2373224 38953516

[33] Zhou W He G Wang H He Y Yuan Q Liu D. Job dissatisfaction and burnout of nurses in Hunan, China: a cross-sectional survey. Nurs Health Sci. (2015) 17:444–50. 10.1111/nhs.12213 26269392

[34] Sun Y Lin MD Liu CM LU Y Liu T Zhao N. To explore the effects of mindfulness-based stress reduction training on job burnout and quality of life of nurses in emergency department. Nurs Res. (2023) 37:3007–11. 10.12102/j.issn.1009-6493.2023.16.028

[35] Salvarani V Rampoldi G Ardenghi S Bani M Blasi P Ausili D. Protecting emergency room nurses from burnout: the role of dispositional mindfulness, emotion regulation and empathy. J Nurs Manag. (2019) 27:765–74. 10.1111/jonm.12771 30887587

